# Implementation of a multimodal home-based rehabilitation intervention after discharge from inpatient geriatric rehabilitation (GeRas): an early qualitative process evaluation

**DOI:** 10.1186/s12877-024-05277-7

**Published:** 2024-08-29

**Authors:** Catharina Roth, Leonie Maier, Bastian Abel, Patrick Roigk, Kilian Rapp, Oliver Schmidberger, Martin Bongartz, Simone Maier, Isabel Wirth, Brigitte Metz, Désirée Immel, Benjamin Finger, Sabine Schölch, Gisela Büchele, Oliver Deuster, Hans‑Helmut Koenig, Sophie Gottschalk, Judith Dams, William Micol, Jürgen M. Bauer, Michel Wensing, Petra Benzinger

**Affiliations:** 1 Department of General Practice and Health Services Research, Heidelberg University Hospital, Heidelberg, Germany; 2 Department of Clinical Gerontology, Robert-Bosch-Hospital, Stuttgart, Germany; 3 Center for Geriatric Medicine, Heidelberg University Hospital, AGAPLESION Bethanien Hospital Heidelberg, Heidelberg, Germany; 4 Geriatric Center Karlsruhe, ViDia Christian Clinics Karlsruhe, Karlsruhe, Germany; 5 AOK Baden-Württemberg Statutory Health Insurance Company, Stuttgart, Germany; 6 Department of Telemedicine, Robert-Bosch-Hospital, Stuttgart, Germany; 7 Institute of Epidemiology and Medical Biometry, Ulm University, Ulm, Germany; 8 Interdisciplinary Centre for Clinical Trials (IZKS) at the University Medical Centre of the Johannes Gutenberg-University Mainz, Mainz, Germany; 9 Department of Health Economics and Health Services Research, University Medical Centre Hamburg-Eppendorf, Hamburg, Germany; 10 Ruprecht-Karls-University Heidelberg, Medical Faculty, Heidelberg, Germany

**Keywords:** Geriatric rehabilitation, E-health, Telemedicine, Post inhouse rehabilitation intervention, Transitional care

## Abstract

**Background:**

Geriatric rehabilitation aims at increasing physical and social activity and maintaining the functional reserve of older people. However, the continuity of geriatric rehabilitation in the outpatient setting is limited due to a lack of structured aftercare programs. In order to overcome this, a three-month multimodal home-based intervention program (GeRas) was implemented. The aim of this early qualitative process evaluation was to assess GeRas in terms of perceived reach, effectiveness/efficacy, adoption/uptake, implementation, and maintenance/sustainability (Domains within the RE-AIM Framework) from the perspective of patients who received the intervention and healthcare providers who were involved in the delivery of the intervention.

**Methods:**

In a qualitative process evaluation, 13 healthcare providers and 10 patients were interviewed throughout the beginning of the implementation period of GeRas to capture early experiences using a semi-structured interview guide. The interview guide and qualitative content analysis was guided by the RE-AIM Framework.

**Results:**

The GeRas program was perceived to be largely well implemented and beneficial by healthcare providers and patients. According to healthcare providers, GeRas showed more advantages compared to usual care. Additionally, outcome expectations were mainly met (Domain 1: Effectiveness). However, the implementation of the intervention delivered via the eHealth system was perceived as challenging (Domain 2: Adoption). Nevertheless, the outpatient physical exercise, the outpatient counselling, and the continuous care after discharge improved perceived well-being regardless of the intervention type (Domain 3: Implementation). To facilitate the continued use of GeRas, technical requirements should be created to increase user-friendliness and to motivate patients to continue the training in the long term (Domain 4: Maintenance).

**Conclusion:**

Although initial experiences with the implementation and effectiveness of GeRas were positive in general, organisational and technical issues need to be resolved to enhance sustainable and successful implementation of the GeRas program.

**Trial registration:**

German Clinical Trials Register (DRKS00029559). Registered 5/10/2022.

## Background

In order to achieve the third of seventeen Sustainable Development Goals of the World Health Organisation (WHO): “*ensure healthy lives and promote well-being for all at all ages*”, rehabilitation is crucial [1]. Limitations in mobility and different activities of daily living (ADL) are particularly prevalent in older adults. These limitations may lead to a loss of independence in this population [2, 3]. The functionality and mobility of older people may be compromised by a decrease in muscle mass, muscle strength, as well as impairments in balance and coordination. In addition, acute illness and hospitalisation increase the risk of mobility loss and precipitate a decline in functionality, increasing the risk of admission to long-term care facilities [4]. Geriatric rehabilitation (GR) plays a key role in maintaining mobility and independence by addressing limitations in daily physical, mental and social functioning due to ageing or acute or chronic illness. Due to financial restraints as well as the lack of human resources and infrastructure in most healthcare systems, access to and duration of GR services is frequently insufficient to reach the declared aim of rehabilitation [5], while the demand is increasing due to the ageing of population [6, 7].

A key element of GR is physical exercise that focuses on improving mobility, endurance, balance, and strength [8–11]. Although research has shown that several months of exercise are necessary to improve balance and strength most effectively [12, 13], the average length of stay in inpatient GR varies from only 7 to 65 days throughout Europe [14, 15]. This is also the case in Germany, where geriatric GR is predominantly provided as an inpatient post-acute program [16]. Although evidence shows that continued exercise at home after discharge from inpatient rehabilitation is effective [17–20], it is usually not offered as a regular program after discharge. Furthermore, geriatric patients require a multiprofessional approach that goes beyond the provision of physical exercise to meet their complex health needs. In order to meet these needs in the context of discharge to the community, close collaboration between patients, caregivers (e.g. family members), healthcare providers (e.g. primary care physicians), and community services (where appropriate) is required to ensure social support and adequate medical care.

In Germany, the responsibility of inpatient settings ends upon discharge, and the organisation of outpatient care is handed over to the patients and their primary care physician. This can lead to gaps in healthcare delivery. As a consequence, health insurance companies are requested to support discharge planning to bridge the gap between inpatient and outpatient care [21]. The discharge process is complicated by organisational factors as the collaboration between inpatient care facilities and health insurance companies oftentimes does not run smoothly. Particularly information sharing across organisations is challenging [22]. In order to overcome communication barriers and enhance information sharing, models of structured collaboration are needed, facilitated by a digital infrastructure. In this context, telemedicine (TM) or telerehabilitation (TR) can be used to overcome communicational barriers between those involved in planning and delivering services to patients and increase the access of patients to healthcare services including rehabilitation [23]. TR as part of the treatment for common conditions found in older people, such as diabetes [24] or frailty, has been proven to be effective [25]. Nevertheless, many older adults and healthcare providers (HCPs) are hesitant to select TR as part of their healthcare [26], and access to TR services is limited. Evidence suggests that one barrier to TR may be that some patients are not comfortable with technology [27]. However, most patients have a positive view of TM once they have used it [28].

To overcome these challenges, a three-month multimodal home-based intervention program following discharge from inpatient GR, the GeRas program, has been implemented at three study centres in Southern Germany. The GeRas program is a multiprofessional intervention that can be delivered either conventionally by home visits and telephone calls (Conventional Intervention Group, CIG) or based on an eHealth system using tablet computers and a combination of home visits and video calls (Tablet Intervention Group, TIG).

Previous research showed that TR programs that have been proven effective failed to be implemented into healthcare practice due to their complexity [29]. Evidence shows that various factors can act as barriers or facilitators of implementing complex interventions, including individual (e.g. lack of personal interest), organisational (e.g. lack of human resources), and healthcare system-related factors (e.g. lack of funding) [30, 31]. In order to optimise the speed and comprehensiveness of implementation, it is crucial to understand these factors [32]. Thus, this qualitative process evaluation aimed to answer the following research question: How did HCPs and patients experience the first phase of the implementation process of the GeRas program, delivered either with or without using a tablet computer, for reach, effectiveness, adoption, implementation, and maintenance?

## Methods

### Study Design of the Main Study

The GeRas program included a three-month multimodal home-based intervention program following discharge from inpatient GR. It is conducted as a three-centre, assessor-blinded, randomized (1:1:1), controlled, parallel-group trial with a three-month intervention period and three-month follow up period. Patients of the intervention arms obtain the intervention either by home visits and telephone calls (CIG) or by an eHealth system using tablet computers and a combination of home visits and video calls (TIG) [33].

### Process evaluation

To evaluate the first phase of the implementation process of the GeRas program, a process evaluation was conducted. The overall aim of this early qualitative process evaluation was to explore how HCPs and participants evaluate the GeRas program in order to identify facilitators and barriers to implementation. The qualitative process evaluation was guided by the Consolidated Framework for Implementation Research [34], a widely used framework in implementation research, and the RE-AIM (Reach, Effectiveness/Efficacy, Adoption/Uptake, Implementation, and Maintenance/Sustainability) framework that was developed to improve the adoption and sustainable implementation of evidence-based interventions (Glasgow, 2019).

### Study setting

The intervention was implemented at three different study sites (I) Robert-Bosch-Hospital Stuttgart, Germany (II) AGAPLESION BETHANIEN Hospital Heidelberg, Germany, and (III) ViDia Christian Clinics Karlsruhe, Germany [33].

### Intervention

The patients in both intervention groups received a three-month multimodal home-based intervention program that aims to improve mobility and social participation. The GeRas program begins upon discharge from inpatient GR, ends after a three-month intervention period, and is delivered by a multidisciplinary team consisting of physical therapists, geriatricians, and social workers based at the discharging GR as well as social workers employed at the statutory health insurance company AOK (German: Allgemein Ortskrankenkasse) Baden-Württemberg [33]. Key components of the GeRas program are (a) an outpatient physical exercise program, (b) outpatient care counselling, (c) person-environment fit (accessibility and adaption of the living environment based on current health conditions of the patient), and (d) nutrition advice. Delivery of the program is monitored by geriatricians, but the medical treatment is left at the discretion of the patients’ primary care physician, who receives a detailed discharge report and records of the patients’ progress. Information sharing between GR hospitals and employees of the health insurance is facilitated by an eHealth system allowing the sharing of documents. Interdisciplinary case conferences, hosted by the eHealth system, take place twice during the intervention period. Participants included social workers, physical therapists, and geriatricians of the study team. The intervention is delivered either via the eHealth systems using tablet computers or in form of home visits and printed training material. Patients in the TIG are provided with a tablet computer equipped with a holder, pen, and a multi-SIM card. The control group receives usual care as well as general health counselling after three months. A detailed description of the intervention can be found in the study protocol [33].

### Implementation activities

In order to promote the successful implementation of the GeRas program implementation activities were conducted and labelled according to the Expert Recommendations for Implementing Change (ERIC) Taxonomy [35] (Table 1). The allocation of the implementation activities to the ERIC strategies was used to facilitate standardisation of research methods and replication.

**Table 1:** Implementation activities applied to enhance the successful implementation of the GeRas intervention

Eric Strategy	Implementation Activities	Format	Participants	Content	Date
Conduct educational meetings	Trainer training	In person	Trainers Clinical study coordination	Procedure of the study Implementation of the training Trainer tasks	September 2022
Conduct educational meetings	Social service training	In person	Clinic social service Insurance social service Clinical study coordination project evaluation	Coordination of the interface between clinic and insurance social service	September 2022
Conduct educational meetings	Clinician training	Online	Clinicians	Procedure of the study Clinician tasks	September 2022
Conduct educational meetings	Software training	Online	Trainers Clinical study coordination Clinical study monitoring Project evaluation Clinical study doctors Clinic social service Insurance social service University Ulm	Handling of the software	Part 1: August 2022 Part 2: October 2022
Conduct educational meetings	Tablet training	In person	Trainers Clinical study coordination Clinic social service Insurance social service	Handling of the tablets	September 2022
Involve patients/consumers and family members	Patient advisory board	In person	Representatives of patients Researchers (clinical study coordination; clinical study doctors; project evaluation)	Part 1: Presentation of the project; Discussion of study material Part 2: Presentation of results of the first meeting; Testing of the training app and tablets Part 3: Optimization of Recruitment; Acceptance of tablets Part 4: to be determined	Part 1: April 2022 Part 2: July 2022 Part 3: May 2023 Part 4: 3. Quarter 2024
Organize clinician implementation team meetings	Trainer workshop	Online	Trainers Clinical study coordination	Discussion of current changes and/ or problems; collegial exchange; ascertain standard procedure	1 meeting per quarter (continuously)

## Study Population

### Healthcare Providers

All participating HCP at the discharging GR hospitals and at the AOK Baden-Württemberg were invited to participate in a semi-structured telephone interview. They had to fulfil the following inclusion criteria to participate in an interview: (I) be involved in the implementation and the delivery of the intervention in one of the three study centres, (II) delivered the intervention to at least two patients regardless of the intervention group, (III) be fluent in German, (IV) older than 18 years, and (V) gave written informed consent.

### Patients

All patients of TIG and CIG included in the main study between October 2022 (start of the main study) and October 2023 were invited to take part in a semi-structured telephone or face-to-face interview. They had to fulfil the following inclusion criteria: (I) have completed the three-month intervention phase of the GeRas program, (II) be fluent in German, and (III) gave written informed consent to the participation.

### Sampling and recruitment

Namey et al. (2016), suggests that thematic saturation may likely be reached at eight to ten interviews when conducting research projects that focus on intervention evaluation [36]. Therefore, the aim was to interview between eight to ten participants per group (HCP and patients) until thematic saturation is reached. The period between October 2022 and October 2023 of the main study represents the first phase of the implementation process of the GeRas program. Thus, the aim was to recruit at least two HCPs of each profession involved. In addition, the aim was to recruit at least eight to ten patients who completed the three-month intervention period between October 2022 and October 2023.

### Healthcare Providers

Envelopes that included an invitation letter, the information leaflet, and an informed consent form for audio-recording the interviews were passed to potential participants by the local project coordinators. Various strategies such as e-mail reminders, online meetings, and the weekly project coordination team meeting were used to maximise the response rate.

### Patients

Between October 2022 and October 2023, patients who received either the conventional or the tablet intervention were invited by post after they had completed the three-months intervention period to take part in the process evaluation. Patients who decided to take part in an interview were requested to contact the process evaluation team directly. To maximise the response rate, patients were reminded by the project coordinator at each study centre via phone.

### Interview guide

The interview guides for both study groups (HCPs and patients) were initially developed by the first author [CR] and were based on the RE-AIM framework. The initial versions of the interview guides were discussed in our department and adjusted accordingly. The interview questions were open-ended and addressed perceptions of HCPs involved in the implementation and delivery of the GeRas program and patients who received either the conventional or the tablet intervention. The interview guides covered among others the following topics: (I) experience with GeRas in general, (II) experience with regards to the work with patients, (III) experiences with regards to teamwork, (IV) evaluation of GeRas, and (V) sustainability and effectiveness of GeRas.

### Data Collection

**Fig. 1:**
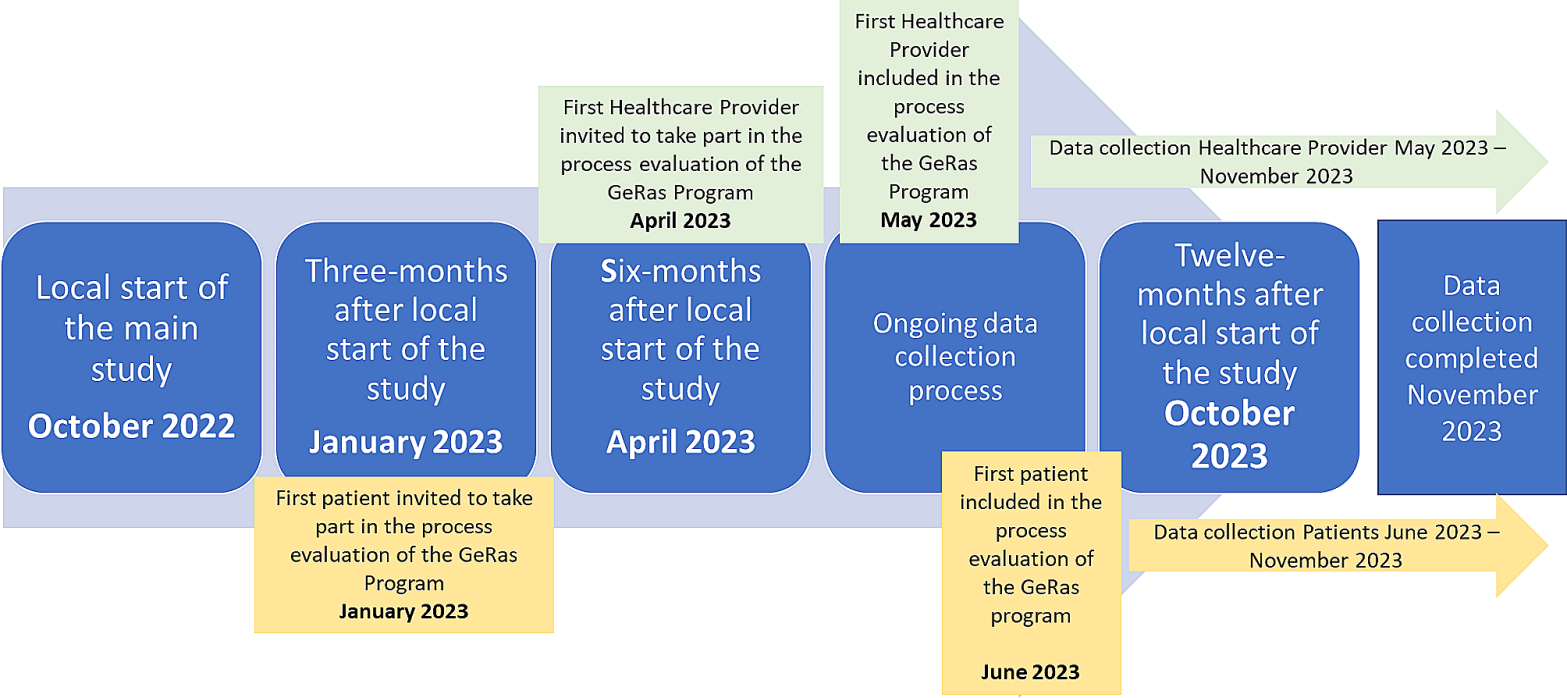
Representation of the data collection process of the qualitative process evaluation of the GeRas program. Green boxes refer to the data collection process of HCPs; yellow boxes refer to the data collection process of patients. The period between October 2022 and October 2023 is considered to be the first period of the implementation process of GeRas, it therefore represents the first experiences of HCPs and patients who participated in the GeRas program

HCP data was collected by a female researcher with a background in health services research and nursing [CR] between May and November 2023 using semi-structured telephone interviews. Each patient was interviewed by a female researcher with a background in health services research and occupational therapy [LM] between June and November 2023. Interviews with patients took place after they had completed the three-month intervention period. The first patients for the main study were recruited in October 2022 and completed the three-month intervention period in January 2023. Thus, the first patients were invited to participate in the qualitative process evaluation in January 2023. The period (October 2022 - October 2023) of the qualitative process evaluation represents the early experiences and perceptions of patients and HCPs with the GeRas program. The data collection process is described in more detail in Fig. 1 (Fig. 1).

All interviews were audio-recorded. In addition, socio-demographic data were collected. Only the interviewer and the interviewee were present during the telephone or the face-to-face interviews. Upon request by the patient, a family member was present for two of the patient interviews. The interviews were pseudonymized and transcribed verbatim. Transcripts were not returned to patients for comment or correction. In order to ensure accuracy, all transcripts were reviewed whilst listening to the audio records by CR and LM. Data collection was finalised after the analysis of existing interview data did not reveal additional new codes, subthemes, or themes which indicated that thematic saturation had been reached. Data is kept in a secure place according to data protection guidelines at the Department of General Practice and Health Services Research.

### Data Analysis

Data was analysed according to Qualitative Content Analysis [37]. After all interviews were transcribed verbatim, the first two authors [CR and LM] familiarised themselves with the whole data set. In step two, the first three interviews (two patient interviews and one HCP interview) were deductively coded based on the RE-AIM framework independently by CR and LM. Additional themes of interest were identified inductively from the data during the analysis. The results were discussed and a final coding system, including deductive and inductive identified codes, was developed [37–39]. The transcripts were then coded line-by-line by CR and LM independently. The coded transcripts were compared against the coding system in further discussions and disagreements regarding codes were resolved (involving a third senior researcher if necessary). The final coding system, including themes, subthemes, codes, and illustrative quotes, was discussed between CR and LM to ensure consensus. Interview data was analysed using MAXQDA version 2020.1.0 [40]. Quotations presented in this paper were translated into English and slightly adapted to maintain cultural meaning by CR (fluent in German and English) and checked for accuracy by LM (fluent in German and English).

### Ethical approval

The study has been approved by the local ethics committees at each study site (Heidelberg: Ethics Committee of the Medical Faculty of Heidelberg University [approval # S-395/2022]; Stuttgart & Karlsruhe: Ethics Committee of the State Medical Association Baden-Württemberg [approval # B-F-2022-057]). Research in this study was conducted in accordance with the Declaration of Helsinki. The study was reported according to the Consolidated Criteria for Reporting Qualitative Studies (COREQ) checklist for qualitative research [41].

### Quality of Data Management

To enhance the credibility of findings rigorous procedures were implemented: (a) using more than one data coder during data analysis, (b) peer debriefing (qualitative research colloquium), (c) consensus discussion between the two coders, and, if necessary, a senior researcher, and (d) member checking with a senior researcher.

## Results

In total thirteen telephone interviews with HCPs were conducted (13 out of 28 HCPs who were initially invited participated in an interview (respondents’ rate 46.4%)). Six patients were interviewed face-to-face and four by telephone (10 out of 56 patients who were initially invited and had completed the three-month intervention period (between October 2022 and October 2023) participated in an interview (respondents’ rate 17.9%). A detailed description of the study population can be found in Table 2.

**Table 2:** Description of the study population

*N* = 23 (100%)	HCPs *n* = 13 (56.5%)	Patients *n* = 10 (43.5%)
Age in years (Median (SD))		
	37 (11.1)	79.5 (4.5)
	Min 25 Max 61	Min 75 Max 91
Gender n (%)		
Identification as female	11 (84.6)	6 (60.0)
Identification as male	2 (15.4)	4 (40.0)
Study Site n (%)		
Site 1: Stuttgart	5 (38.4)	4 (40.0)
Site 2: Heidelberg	5 (38.4)	3 (30.0)
Site 3: Karlsruhe	3 (23.2)	3 (30.0)
Profession n (%)		
Physical therapists	4 (30.8)	Not applicable
Geriatrician	2 (15.4)	
Social Worker employed at discharging GR	2 (15.4)	
Social Work employed at Health Insurance company	5 (38.4)	
Data Collection Method n (%)		
Face to Face Interviews	0	6 (60.0)
Telephone Interviews	13 (100)	4 (40.0)
Work experience in Years (Median, SD)	16 (8.9) Min 1 Max 33	Not applicable
Intervention n (%)		
Tablet Intervention	Not applicable	3 (30.0)
Conventional Intervention		7 (70.0)
Interview Duration (Median) in minutes	26.4	30
	Min 21.28	Min 22
	Max 42.4	Max 54

### Results of the qualitative content analysis

Four domains were deductively identified based on the RE-AIM framework. Each domain included several inductively identified themes: **Domain 1 Effectiveness/Efficacy**: Expectations and perceived outcomes related to the GeRas program, **Domain 2 Uptake/ Adoption**: Tablet vs. Conventional Intervention, **Domain 3 Implementation**: Evaluation of the different components of GeRas, and **Domain 4 Maintenance**: Scalability and Sustainability of GeRas. Exemplary quotes were used to illustrate the meaning and themes identified during analysis. Quotes were anonymised to protect the identity of HCPs and patients who participated in an interview. The **Domain Reach** refers to the absolute number or proportion of individuals who were willing to participate in the GeRas program and reasons why or why not. The recruitment to participate in the main study has not yet been completed, data on Reach will be therefore be published elsewhere.

## Domain 1: Effectiveness/Efficacy (Table 3)

**Table 3:** Domain 1 Effectiveness/Efficacy: expectations and perceived outcomes related GeRas

Effectiveness/ Efficacy	This domain describes the impact of GeRas on an individual level, including negative effects and broader impacts such as quality of life or economic outcomes from the perspective of HCPs and patients.
Theme*	**Definition**
General Evaluation	This subtheme describes how HCPs and patients overall evaluated the intervention.
Outcome Expectancies	This subtheme includes statements related to outcome expectancies related to the GeRas Program
Perceived Outcome	This subtheme includes statements related to perceived outcomes achieved through participating in the GeRas Program
Expectations Regarding Healthcare Delivery	This subtheme describes Expectations HCP had related to the GeRas Program

*all themes were addressed by both study groups; HCPs: Healthcare providers

### General evaluation

GeRas was evaluated as beneficial by HCPs and patients aside from technical challenges. They were all satisfied and stated that GeRas had some advantages compared to usual care. HCPs mentioned that they saved time because documentation was made easier by the health system used in GeRas. Moreover, travel time was saved because home visits were replaced by video consultations.

[…]home visits require more time not only due to journey time but actually you have a lot more distractions and other topics during a home visit, of course that requires more time spent on site compared to an in-depth video consultation, which is supposed to replace the home visit for the tablet group. From a health economical perspective, the eHeatlh aspect is to be emphasised […]. (Physical therapist, Female, Age 43)

No, that was fine. It was really fine, I was satisfied. (Patient, Male, Age 76)

### Outcome expectancies

HCPs and patients had various expectations of GeRas. Patients expected the outpatient physical exercise program to maintain or improve their mobility. HCPs were able to confirm these statements. They also expected the training to increase the patients’ mobility, but also their independence, and quality of life.

I had the expectation that it would get better with all the exercise. Because I do get physiotherapy, but only once or twice a month, a week at the most. And of course, you do it, but it’s not as intensive as when I do it for 10 min every day. I am forced to do it [due to the GeRas]. And that’s why I had the expectation that this program would actually help. And I think it did help, I think it did. (Patient, Female, Age 91)

[…] I had very positive expectations, the expectation that I can help people with my work and support them to actually become more independent again, yes, that was the greatest expectation. (Physical therapist, Female, Age 39)

### Perceived outcome

The majority of patients stated that their expectations had been fulfilled, especially since their mobility had improved through continuous training. Patients also perceived progress in their independence and quality of life. HCPs shared this experience.

I say yes, I have achieved what I wanted. I can move freely again. Except that I’m still a bit limited, but that has to do with the fracture and nothing with the exercises. Before, I couldn’t walk at all. I was lying in bed or had to get up with difficulty […] and now I can do all that without any problems. (Patient, Male, Age 79)

That’s how it is, and I have to say honestly whether it was tablet or conventional, you saw, for some very quickly, after a week, but after three to four weeks you really saw an increase in quality of life. (Physical therapist, Female, Age 52)

### Expectations regarding Healthcare Delivery

Most HCPs stated that GeRas would improve their daily work with older people in general and support them to regain independence. Some HCPs mentioned that particularly the TM/TR component was of interest to them. They expected it to work without technical difficulties. This expectation, however, could not be fulfilled which left most HCPs disappointed.

I actually had bigger expectations towards the technical possibilities, sadly it was a bit disappointing, that technical details were not sorted out, the platform had frequent technical errors […] we practically could not use the tablet, which makes it difficult and also a little disappointing, because I was very curious about the concept itself and thought that it had potential. (Social worker of the health insurance company, Female, Age 28)

## Domain 2: adoption (Table 4)

**Table 4:** Domain 2 adoption: tablet vs. conventional intervention

Adoption	This domain describes how HCPs and patients evaluated and accepted GeRas delivered either with or without telemedicine.
Theme*	Definition
Evaluation of the Tablet Intervention	This subtheme describes how patients and HCPs evaluated the tablet intervention overall.
Technical Challenges	This subtheme describes technical challenges that had an impact on the delivery of the tablet intervention during the implementation.
Evaluation of the Conventional Intervention	This subtheme includes statements that describe experiences with regards to the conventional intervention.

*all themes were addressed by both study groups; HCPs: Healthcare providers

### Evaluation of the tablet intervention

Overall, patients assigned to the TIG were satisfied with GeRas except for technical challenges that occurred at the start of the implementation phase. They evaluated the training videos as practical. HCPs were able to confirm these statements. They stated they were satisfied with the tablet intervention if technical requirements were fulfilled and the intervention could be delivered as planned.

I liked that they did the exercises very expressively. […] they showed it correctly and yet that was all good. And when you sit down, how you take your arms and so on, it was already very well thought out. (Patient, Female, Age 91)

[…] I had mainly good experiences with the tablet intervention quite well, if there are no technical difficulties, the patients are very motivated […]. (Physical therapist, Female, Age 28)

Apart from technical problems at the start of the implementation phase, the user interface was rated positively by HCPs and patients. Particularly, the user interface designed for patients was described as intuitive and easy to understand.

Overall, I think the user interface for the patients exceeds my expectations, I think the user interface looks very good, and it is very easy to use. I am really surprised how well the provider has managed to adapt it for the target group. Of course, I don’t know how the target group experiences it, but I think it’s very well done and scalable […] I am very satisfied. (Geriatrician, Female, Age 49)

A key challenge at the beginning of the implementation phase was the occurrence of technical problems with the eHealth system and the tablets provided for patients. These problems made it difficult, to deliver the intervention as planned. Patients described that temporarily they were not able to conduct or receive video calls due. Some patients perceived it as time-consuming to re-watch the training videos over and over again since they remembered the exercises and did not need the tablet computer to perform them. HCPs stated that they had the impression geriatric patients need physical and personal contact to assist with the technical challenges of TM/TR. They mentioned that future generations may benefit more from TM/TR intervention compared to the generation included in the presented study.

Well, I tried to get rid of the technical issues, I kept rebooting to get to it, I also said that, then another colleague switched on, then we could make video calls and it worked two or three times and then it crashed again. […]. (Patient, Female, Age 75)

And maybe in one or two generations you know the people who are relatively young nowadays, in later age of course they can handle it. But still with the tablet computer, I have to say, people are used to this physical contact, whether it’s physiotherapy or massage or lymphatic drainage, depending, that’s the disadvantage I see with the tablet computer, there’s no physical contact […]. (Physical therapist, Female, Age 52)

HCPs stated that the main advantage of the tablet intervention compared to conventional intervention was that patients were able to demonstrate the exercises via video and physical therapists could correct them or give general advice. Physical therapists additionally stated that this made it easier to increase or decrease the level of exercise intensity according to the current fitness level. Another advantage was that weekly video consultations were perceived as a closer and more intensive contact by physical therapists compared to a usual phone call. Some HCPs also mentioned the tablet intervention as helpful to reach patients living in rural areas. In addition, a video consultation would be more time-efficient.

Exactly, so if the […] internet connection was stable, then we could actually use the tablet computer to show exercises or have them show us exercises, which is the big advantage compared to a normal telephone call and actually also to talk about the performance of the exercises, apply corrections on the video, […] when pain occurred the patients could not only describe it but also show it. That was actually such a big advantage […], on the part of the patients with whom we successfully carried out video consultations, it was more personal, […] I found that all positively compared to a normal telephone call, you simply had better access to the patient and could also simply demonstrate something from the therapist’s side, I found that to be a very big advantage. (Physical therapist, Female, Age 43)

### Technical challenges

The start of the implementation of the tablet intervention was perceived as challenging, due to the need for certain technical requirements, by HCPs and patients. Both groups described technical challenges of various kinds. Technical problems included, for example, videos that could not be played fluently, lack of Wi-Fi access, connection problems during video calls, software crashes, or unexpected logout of the software. The main problem was the need for a stable internet connection to carry out the intervention as planned. The project team together with the software provider were able to resolve most of the technical issues that challenged the implementation of the tablet intervention at the beginning.

At the beginning, I would say, it was the technical problems that came up, but a lot of things have been solved in the meantime and are running well, or we now know how to do things better […]. (Physical therapist, Female, Age 39)

[…] because the tablet computer kept crashing, or I got something completely different on the display […]. (Patient, Female, Age 75)

### Evaluation of the conventional intervention

The main advantage perceived by patients was the visual illustration of the physical exercise through the provided training poster. They explained that the visual illustration helped them to perform the exercise correctly. HCPs confirmed this experience. They mentioned that they had the impression that the visual illustration helped patients stay focused and motivated. The visual illustration through the training poster was a big difference to the tablet intervention group where patients actively needed to start the tablet computer to perform their training.

[…] So the advantage for me was that I practically had the poster where I could proceed exactly according to […]. (Patient, Female, Age 82)

So the big advantage of the poster was actually that the patients simply had this poster in front of them, [… where they really looked at it every day, which was not necessarily the case with the tablet computer, where you really had to actively go and use the tablet, and I think that was the big advantage with the poster patients, that they always had the exercises in front of their eyes in their daily routine and were automatically reminded of them more often, […]. (Physical therapist, Female, Age 43)

HCPs felt that the main advantage of the conventional intervention was the personal contact. Personal contact was perceived as motivational and more intense compared to video consultation. This impression was confirmed by patients’ statements. Nevertheless, this experience was not shared by all HCPs.

I feel it is a better accompaniment because I see the patients at home and can simply do the exercises with them at home […]. And the personal contact, I have the feeling that this is more intense for many patients and also, yes, a different commitment […] compared to the tablet group […]. (Physical therapist, Female, Age 39)

One big disadvantage of the conventional compared to the tablet intervention was that to increase the level of the exercises, HCPs had to wait for the next scheduled home visit. They highlighted that they did not feel safe to increase the level via telephone without actually being able to see and control whether patients were capable of performing the next exercise level.

It was actually the same phone call [like the tablet group], except that we did not see each other, which made it a bit more difficult to adapt the exercises because we could not show them how to do it. On the tablet computer, if we changed something, for example, the level of the exercise, the patients saw it on the tablet computer the next day at the latest. The poster group did not see that. […] That means […], I only increased the level when we saw each other.[…]. (Physical therapist, Female, Age 28)

## Domain 3 implementation (Table 5)

**Table 5:** Domain 3 implementation: GeRas, evaluation of the different components of the program

Implementation	This theme describes how HCPs and patients evaluated the three-month multimodal home-based intervention GeRas based on the different components.
Theme*	**Definition**
Outpatient Physical Exercise Program	This subtheme includes statements regarding experiences regarding the implementation of the Outpatient Physical Exercise Program.
Outpatient Care Counselling	This subtheme includes statements regarding the experiences of the implementation of the Outpatient Care Counselling
Nutrition Advice	This subtheme includes statements regarding the implementation the experiences and acceptability of the Nutrition Advice
Interprofessional/ Interdisciplinary Teamwork	This subtheme includes statements related to interprofessional/interdisciplinary teamwork.
Continuous Care after Discharge	This subtheme includes statements regarding the benefits of the continuity of care after being discharged from the rehabilitation clinic due to the implementation of the intervention.
Home Visits	This subtheme includes statements regarding the Home Visits that are part of the intervention.
Regular Check-Up-Phone Calls	This subtheme includes statements regarding the regular Check-Up-Phone Calls that are part of the intervention.
Suggestions for Improvements	This subtheme includes suggestions for improvement.

*all themes were addressed by both study groups; HCPs: Healthcare providers

### Outpatient Physical Exercise Program

The physical exercise program was evaluated positively by all patients regardless of the intervention group. Particularly the outdoor walk within the patients’ neighbourhood as part of the exercise program was perceived as beneficial and motivational by most patients. However, some patients described that the exercises at the beginning of the intervention phase were too difficult and the level needed to be adapted. HCPs could confirm these experiences. They mentioned that particularly the outdoor walks increased independence and were beneficial for all patients. The exercise that aimed at strengthening the lower limbs including using stairs, however, was perceived as difficult to perform for several reasons. Nonetheless, HCPs perceived the adaptability of the physical exercise program, especially the opportunity to modify the degree of difficulty according to the physical capacity of participants as a key advantage of GeRas. Patients confirmed these perceptions and stated that adapting the degree of difficulty or decreasing/increasing the number of sets according to their current state of health helped them to complete the training.

And then during the three months intervention period, an increase is also possible, but that’s what I like about GeRas for example, that it is individually adaptable. The degree of difficulty can be adjusted in both directions, […] if you notice that there might be some regression or some impairment that makes it necessary to adjust the exercises to make them easier, that this is also possible. (Physical therapist, Female, Age 39)

Yes, that always came up, now just when I had cramps again or so, then I just said, I’m sorry I cannot do the exercise properly or I cannot go back and forth seven times at a set, I will just do it four times now or even when getting up from the chair five times. I did that three times at first and then just increased that. And I am now at a very normal level. (Patient, Female, Age 82)

### Outpatient Care Counselling

Almost all patients evaluated the outpatient care counselling offered by social workers of the health insurance as beneficial. They valued the support and the information they received as mainly positive. HCPs backed these experiences and indicated that although some patients were well informed, for most of them outpatient care counselling was useful and identified further needs. HCPs reported that direct contact with patients by social workers from the health insurance increased patients’ satisfaction with healthcare services and made them feel adequately cared for.

The outpatient care counselling of the health insurance fund. I was awarded long-term care funding through that. Then I got the outpatient nursing service because of that and it works wonderfully. They also recommended Meals on Wheels service, but I didn’t take it. I took the one I had before. The one they recommended cost 8 Euros and at the other one meal costs around 5 Euros. And that works wonderfully. (Patient, Male, Age 79)

So I think it definitely made sense for the patients. As I said, there are also the independent ones who are well informed already, but I would say that for two thirds it was definitely useful, […]. And as I said, they [the patients] felt well looked after, I had the impression that they felt like they were taken care of […]. (Social Worker of the health insurance company, Female, Age 36).

### Nutrition advice

Only a few HCPs mention the nutrition advice as part of GeRas, and only one patient talked about it at all. Although HCPs perceived giving nutrition advice as useful, they indicated that it was not entirely clear who was responsible for it. HCPs also stated they did not feel competent enough to issue nutritional advice.

[…] the only thing I did not need was nutrition advice. My wife always cooks for us, or we cook together. I do not have any problems related to nutrition […]. (Patient, Male, Age 76)

What I didn’t find good or optimal was the nutrition advice, which doesn’t really take place from our side, but we practically record the current state, i.e. how the nutrition is at the moment, and unfortunately, we don’t know that much about it, I have to say. Exactly, and that was a bit problematic, exactly, that means that the insurance company would probably have to intervene because they have their people for that, their experts. (Physical therapist, Female, Age 28)

### Interprofessional/ interdisciplinary teamwork

HCPs, regardless of their profession, talked positively about the interdisciplinary case conference. The opportunity to share information and identify gaps in patients’ healthcare was perceived as a benefit compared to usual care. Sharing healthcare-related information between social workers of GR and health insurance employees was perceived as an advantage for patients. HCPs explained that due to a structured handover, loss of information could be prevented which lead to well-organised discharge management.

That may sound a bit terse at first, but I think this information is also passed on in our team meetings. Then the AOK can check on the participant again, “is there perhaps an offer where the persons can meet other senior citizens or get a warm senior citizens’ lunch” so that there can also be an exchange of information again, maybe the person is lonely or cognitively declining, maybe it has to be discussed again somehow with the family doctor whether there is a change that is noticeable, so yes, it is very useful. (Social Worker, Female, Age 28)

### Continuous care after Discharge

In addition to the physical exercise program and the outpatient care counselling, which was described as very positive overall, the continuity of care after discharge from the inpatient GR was seen as the key to successful aftercare by both patients and HCPs regardless of their profession. Most patients described that the professional support after being discharged from inpatient GR facilitated their recovery and contributed to their well-being.

I think it [GeRas] should continue because the patients simply have a chance to continue to be connected to care, of course the patients are always connected to further outpatient care through their GP practice, but I have the impression that the transition to outpatient care runs more smoothly when they are accompanied by us. (Social Worker, Female, Age 28)

Exactly, so you’re in rehabilitation, after discharge you’ve done a lot for your mobility. You then feel much fitter. And then, over time, you learn the appropriate gaits, the walking routes, where you can do things. Where you can do things for yourself again and again. And then that falls away. And if you have an appropriate program, like now or like me, then that’s a positive thing. Because you’re lazier on your own. You don’t just do so much anymore. (Patient, Male, Age 76)

### Home visits

Patients described the home visits by the different HCPs as very supportive. Especially due to the limited mobility, home visits were perceived as useful. HCPs shared this experience. They mentioned that the scheduled home visits as part of GeRas revealed gaps in healthcare, by receiving an on-site impression of the home environment. These gaps could then be discussed with the multiprofessional team to improve holistic healthcare.

I thought that was great too, that’s why I signed it straight away because I thought I would have it [the training program] in the house and wouldn’t have to go into town, because I wouldn’t have been able to walk so well anyway and would have needed someone to drive me all the time. And that was unnecessary, wasn’t it? […]. (Patient, Female, Age 87)

[…] the home visits, […] from the point of view of a social worker, a home visit also activates people because it is a visit that takes place calmly […] it activates the people and issues also come to light, such as that the post box is totally overflowing, I say, or yes, patients cannot be reached because the landline phone is not working. (Social Worker, Female, Age 28)

### Regular Check-Up-Phone calls

The regular check-up-phone calls were evaluated ambivalently. Although the majority of patients appreciated the calls, some stated that they were unnecessary compared to a home visit. HCPs did not agree on the necessity of the phone call. Some felt that phone calls were just as valuable as home visits, while others preferred home visits to phone calls or video calls to phone calls.

[…] As I said, I was happy when she called today… But, yes, that I hear her again, that’s like when a friend calls. But, yes, so if she was here in person and could check and say, you’d better do it like this now and try it again or something. That’s different, isn’t it? You can’t convey that from a distance over the phone. (Patient, Female, Age 87)

I don’t think so, so I’ll put it this way, I’ve also had very good experiences with telephone calls, so I wouldn’t say that a telephone call is always worse or has to be inferior in quality to a home visit. I think a well-conducted phone call certainly has the same value as a video call. (Social Worker of the health insurance company, Female, Age 28)

### Suggestions for improvement

HCPs mentioned the importance of adapting the program to the current healthcare needs of patients. The number of home visits, check-up-phone calls, or video consultations could be adapted in order to protect patients and their relatives from being overloaded due to a busy schedule. Patients backed this statement, some of them mentioned that they would prefer a more individualised approach based on their current healthcare needs. Patients stated that although the outpatient physical exercise program was perceived as beneficial, including training sessions to improve strength in the upper body and the arms would increase the quality of GeRas. Moreover, almost all patients mentioned that the number of accompanied walks by physical therapists during home visits was insufficient and should be increased.

And I would be more needs-oriented, which means really looking at every aspect, is every contact needed, are two, three, four, five maybe more needed. Is the nutrition advice needed, is the social service needed, is the physiotherapy needed? To not have that rigid guideline, but rather having certain offers and maybe seeing them more as elements, like a program I can individually put together […]. (Social Worker of the health insurance company, Female, Age 36).

That would perhaps be the point where you could say, well, you could make a little more progress. Yes, the main thing now is to focus on balance and more or less on the legs, on the strength in the legs. And maybe also doing a bit more for your arms. But I’ve actually done that for myself. That works too. (Patient, Male, Age 76)

## Domain 4: maintenance: scalability and sustainability of GeRas (Table 6)

**Table 6:** Domain 4 maintenance: scalability and sustainability of GeRas

Maintenance	This theme describes how HCPs and patients assessed suitability, sustainability, and scalability of the three-month multimodal home-based intervention GeRas.
Theme*	**Definition**
Suitability	This subtheme describes the suitability of the intervention from the perspective of HCPs and patients that delivered or received it.
Sustainability	This subtheme how HCPs and patients assessed the sustainability of the intervention.
Scalability	This subtheme describes how HCPs and patients assessed the scalability of the intervention.

*all Themes were addressed by both study groups; HCPs: Healthcare providers

### Suitability

HCPs explained that GeRas was not suitable for everyone. Although inclusion/exclusion criteria for the main study were strict lack of cognitive and physical abilities played a significant role regarding the suitability of patients to successfully participate in the GeRas program. In addition, HCPs mentioned that some patients’ lack of interest in health promotion measures such as home training had an impact on the suitability to participate in the intervention regardless of the group. They stressed that GeRas was only suitable for patients who were interested in improving their health. Patients somewhat backed this statement. They indicated that motivation played a significant role but also the social environment and social support needed to be considered regarding suitability. HCPs also mentioned that GeRas worked better for patients who were supported by relatives. Most of the HCPs stated that the age group included in this study did not have sufficient affinity for technology. They agreed that GeRas would work better in a few years.

My suggestion would be to look again at who is really suitable for which group and who already has the physical abilities to do these exercises, I think that is actually another point where you would have to look more specifically at who is suitable […]. (Social Worker of the Health Insurance Company, Female, Age 28)

Who would then also be a bit motivated and not just complain, you know, because they are alone. I cannot say that about myself. I’m motivated because I still want to, but also because my social environment is right. For example, previous Saturday I was with my granddaughter, grandson and her boyfriend, we had breakfast and so on. They come and pick me up […]. (Patient, Female, Age 79)

### Sustainability

Some patients mentioned the need for sufficient funds to implement GeRas sustainably. Funding was also mentioned related to a sustainable implementation of GeRas by a few HCPs. In addition, HCPs agreed on the need to have a sufficient number of healthcare professionals available to deliver the intervention correctly. Almost all HCPs explained that the main reason for a sustainable transfer of the intervention into standard care was the added benefit for patients. GeRas made it possible to identify gaps in healthcare, to improve the quality of life of patients, and to meet the need for professional support after discharge. Almost all patients also stated that the continuity of care after discharge was the main reason for them to transfer the intervention into standard care.

Yes, you also need money. But as I said, from a cost-benefit analysis - I did economics once - I can only say that it’s worth it. It’s a good investment. (Patient, Male, Age 80)

Yes, so it contributes a lot. For someone who doesn’t do anything, it’s of course a huge step forward. I have always done something for myself. I was also motivated, but for someone who is new to it, I could imagine that it would have a really positive effect […]. (Patient, Male, Age 76)

And if necessary, one would also have to look at the physical therapists who make home visits, that sufficient resources are also made available, explicitly sufficient time and travel allowance to visit people who live in the countryside. (Social Worker, Female, Age 28)

### Scalability

According to most HCPs, one hindrance that may affect scaling GeRas up were probably technical requirements. HCPs explained that technical requirements were currently not fulfilled outside of GeRas and negatively influenced the implementation. Nevertheless, HCPs found that GeRas was a very useful intervention and would be worth implementing sustainably and on a large scale. Moreover, it would make sense to extend the GeRas concept not only to the geriatric sector but also to other areas. Including other study populations, e.g. focusing on people that lack German language proficiency could also be useful. They would also benefit from GeRas, especially from tablet intervention, as language barriers can be overcome. Including nursing homes or senior centres was also something HCPs and patients mentioned in order to reach a wider population.

[…] From a sociological point of view, I would also find it interesting to determine the needs of people who do not speak German so well, […] I could imagine that people with a different mother language would quickly learn how to use a tablet if it was not designed in German, but perhaps worked more with symbols, and could generally benefit from this program. (Social Worker, Female, Age 28)

[…] Yes, well, I wouldn’t shy away from pursuing this [GeRas] right into the nursing homes […]. (Geriatrician, Male, Age 61)

Yes, in a nursing home or senior centre. The people who live at home are actually […] reasonably mobile. But especially in nursing homes, when someone really comes to me […]. (Patient, Female, Age 79)

### Summary of facilitators and barriers

In total 12 facilitators and seven barriers that may influence the success of the implementation of the GeRas program were identified from the perspective of HCP and patients (Table 7).

**Table 7:** Facilitators and barriers related to the implementation process of the GeRas program

Facilitators	Barriers
**Domain 1: Effectiveness/ Efficacy**	
- Documentation via telehealth application	
**Domain 2: Uptake/ Adoption**	
- Intuitive, easy to use interface - Personal contact specially to assist with technical challenges - Weekly video consultations with trainers - Regular communication with software provider to assist with technical challenges - Visual illustration of the exercises	- Technical challenges with the application or device - Time-consuming videos that cannot be skipped - Insufficient Wi-Fi connection
**Domain 3: Implementation**	
- Possibility to modify the degree of difficulty for each exercise - Interdisciplinary exchange of information and experiences - Structured handover of information between in- and out-patient providers - Home-visits and personal contact with trainers	- Lack of individualization of the program components to meet individual health needs
**Domain 4: Maintenance**	
- Support by family members or friends - Affinity for technology	- Lack of physical and cognitive abilities - Patients lack of interest in health promotion - Insufficient funding options

## Discussion

According to participants of this qualitative process evaluation the GeRas program was perceived as beneficial regardless of the intervention group (conventional or tablet) by HCPs and patients. The implementation of all program components was perceived as successful by patients and HCPs. In particular, the outpatient physical exercise program as the core component of GeRas was evaluated positively by all interviewed HCPs and patients. Technical challenges, however, were perceived as the main hindrance impeding the implementation of the GeRas program within the eHealth system-based intervention group.

With regard to *Effectiveness* (Domain 1), outcome expectations of patients and HCPs were met and the perceived effectiveness of the program was confirmed by HCPs’ and patients’ reports. Patients as well as HCPs noticed improvements in mobility, quality of life, and social participation, verifying the presumed objective effects of aftercare programs in GR [19, 20]. However, these perceptions must be confirmed by means of the outcome evaluation of the main study [34].

Regarding the *Adoption* (Domain 2) of the GeRas program, the TR component was perceived as a challenge by HCPs and patients. Challenges emerged mainly due to technical requirements that sometimes could not be fulfilled predominantly during the initial stage of the implementation phase. Apart from a generally unstable internet connection, the telehealth application showed a few technical difficulties, especially during the initial intervention period. In addition, HCPs emphasised that the age group included in this study was highly challenged by dealing with TM/TR measures and needed personal contact with their HCPs. This seems to be a frequent barrier in studies evaluating geriatric telehealth programs similar to the GeRas program [27]. Although a lack of technical skills in geriatric patients has previously been identified as a barrier in a systematic review conducted by Batsis et al. [42], HCPs also showed pre-existing prejudices towards geriatric patients which have been proven to act as a barrier to implementation. Benefits of TR, summarized in a review conducted by Hayes [43], included for example, reduced travel times and more efficient use of resources [43]. These benefits were also reported as a potential effect of the GeRas intervention by HCPs and patients but were not experienced explicitly during the initial intervention period. This may be due to the initial house visit HCPs provide regardless of the intervention group. Nevertheless, if technical requirements were met and the intervention could be carried out as planned, it was perceived as beneficial. As discussed in previous research conducted in the United Kingdom (UK) on the experiences of delivering care remotely among practitioners in a UK geriatric medicine clinic, one major disadvantage of the telehealth intervention seems to be the omission of physical touch and body language [44]. HCPs of this study reported that lack of physical touch limited their abilities especially when working with physically more impaired patients, making the telehealth intervention less applicable for these patients. As shown in the systematic review by Batsis et al. [42], physical touch and personal contact through healthcare professionals seem to be an important facilitator during the implementation of telehealth [42] .

Overall, the *Implementation* (Domain 3) was perceived as successful, although the eHealth system component of the program faced some challenges. HCPs found the home visits especially important to establish a trustful therapeutic relationship with their patients. Components such as weekly phone or video consultations were evaluated more ambivalently. The interview data showed a high need for social interactions which may be caused by the social isolation frequently experienced by geriatric patients according to an umbrella review by Collado-Mateo et al. [45]. The GeRas program seems to compensate for social isolation by providing regular social contact in the form of phone calls, video calls, or home visits. Compared to phone calls, weekly video consultations were perceived as more useful, especially by HCPs, as they were able to see patients and visually control the execution of exercises. This perception contradicts previous findings which proposed a lack of visual control to be one of the main disadvantages of telehealth interventions by practitioners in a UK geriatric medicine clinic [44]. The initial personal contact included in the GeRas project seemed to be an advantage in comparison to similar projects based solely on telehealth [42].

HCPs and patients perceived the organised discharge management from inpatient GR to the home environment and the continuous care after discharge (the three months intervention period) as particularly beneficial. This underlines the demand for structured discharge management programs within GR in order to maximise the effects of rehabilitation and minimise risks of rehospitalisation [12, 13]. HCPs emphasised the importance of adaptability of the program components in light of the complex, individual health needs of patients. A study conducted in a municipal visitation unit in the Northern Denmark Region also concluded that individual needs must be considered during intervention design especially the complex needs of geriatric patients [46]. The interdisciplinary collaboration was also perceived as a key advantage of the GeRas program by HCPs. This confirms the findings of a previous study conducted in Germany, stating that in order to meet the complex health needs of geriatric patients, a multi-professional approach is required [47]. Although the GeRas project provides multi-professional support, future programs may also consider the integration of other healthcare professions, such as occupational therapists, nutritionists, nursing specialists or pharmacists, who are equally relevant to geriatric care.

Some HCPs indicated the limited financial and human resources available for GR as the main factor hindering sustainability and *Maintenance* (Domain 4) of the GeRas program after the project completion. This is in line with recent research demonstrating that the unavailability of resources was a frequent issue in the implementation of rehabilitation programs [29]. This barrier could be omitted by working closely with Health Insurance Companies such as the AOK in order to guarantee funding. HCPs and patients emphasized that suitability and patient characteristics played an important role in the success of the implementation of the GeRas program. A systematic review conducted by Everink [10], revealed similar factors that have been shown to influence the possibility of home discharge and maintenance of exercise programs, especially for geriatric clientele [10]. Nevertheless, HCPs acknowledged the positive effect of the program and advocated for its maintenance and implementation into standard healthcare after the project’s completion.

In conclusion, this early process evaluation showed that a multimodal home-based rehabilitation intervention after discharge from inpatient GR can help to ensure rehabilitation success. This reinforces the feasibility and acceptability of telehealth by older patients if certain aspects, such as adaptability and patient factors are considered [42]. The choice of patients who can participate in the TM/TR intervention should be carefully evaluated before inclusion to prevent them from terminating the program prematurely due to technical challenges or physical or cognitive barriers.

## Strengths and limitations

Semi-structured interviews in combination with rigorous qualitative analysis were considered an appropriate research approach to answer the research questions of the study. A key strength of this study was the exploration of shared and/or contradictory experiences of HCPs and patients included in the study. The methodological approach made it possible to identify barriers and facilitators from the perspective of all parties involved in the GeRas program. In order to reduce the risk of losing content, data analysis was guided by standardised methodological procedures, and the COREQ checklist was used to guide reporting qualitative findings [41].

Some limitations have to be acknowledged. Although experiences were relatively consistent among the study participants, a limitation is that these findings may not be transferable to other contexts, patients, or HCPs. For example, the results of this study may not be transferable to countries with an overall stable network coverage or countries that are experienced in dealing with TR interventions with geriatric patients. A further limitation to consider is the “locality” in that the German health system setting and specific national and regional factors may have influenced results. In particular, segregation between inpatient and outpatient care with lack of established co-operation clearly shape the expectations of participating HCPs and patients as well as their experiences with the GeRas program. The role of health care insurances in supporting their members with organisational issues related to post-discharge care is due to this lack of established cooperation between providers in the German health care system. At the same time, German health insurances are offering mandatory long-term care insurance as well, raising their interest in preventing the need for long-term care. This explains the employment of social workers by health insurances for counselling of their members. In addition, participants who voluntarily participated in an interview might have different experiences compared to HCPs and patients who chose not to participate in an interview. For example, patients who did not participate may perceive fewer or no barriers or completely different aspects that may facilitate or enhance the implementation of the GeRas program. Social desirability may have influenced the participants’ responses.

At some phase during the analyses phase no new codes, subthemes, or themes were identified, which indicated that data saturation had been reached. Nevertheless, a higher number of HCPs and patients may have led to more diverse results. As the quotes were translated from German into English, it is possible that the meaning of the translated quotes differs to some extent from the original meaning in German. Interviews with both study groups were conducted during the first period of the implementation process of the intervention, challenges that may arise in the early phase of a project may have influenced the participants’ answers; thus, later interviews might lead to different results. The results of the study must be interpreted with caution in terms of generalization and representativeness due to its qualitative approach.

## Conclusion

Although the initial experiences with the GeRas program were generally positive, organisational issues were highlighted by both study groups that need to be addressed to improve successful implementation and sustainability. In particular, the usability of TR provided via tablet, e.g. stable and powerful mobile internet, needs to be improved to make the program more user-friendly and to maintain motivation to carry out the physical exercises in the long term. The results of this early qualitative process evaluation suggest that it is possible to accompany geriatric patients after inpatient GR using TM and to ensure rehabilitation success if technical requirements are fulfilled. Nevertheless, it became apparent that TM/TR is not an option for all geriatric patients and that social contact with HCPs is mandatory. A mixture of TM/TR and home visits appears to be the best solution for this population. Additionally, revealed the process evaluation, that to transfer the GeRas program into standard healthcare sufficient financial and human resources are needed. This perception, however, needs to be verified by the economic analysis.

## Data Availability

The datasets generated and analysed during the current study are not publicly available due German data protection law but are available from the corresponding author on reasonable request.

## Abbreviations

ADL: Activities of Daily Living
AOK Baden Wuerttemberg: Statutory Health Insurance Company Baden-Wuerttemberg (German: Allgemein Ortskrankenkasse Company Baden-Wuerttemberg)
COREQ Checklist: Consolidated Criteria for Reporting Qualitative Studies Checklist
ERIC: Expert Recommendations for Implementing Change
GeRas program: Three-month Multimodal Home-based Intervention Program
HCP: Healthcare Provider
RE-AIM Framework: Reach, Effectiveness/Efficacy, Adoption/Uptake, Implementation, and Maintenance/Sustainability Framework
TM: Telemedicine
TR: Telerehabilitation
UK: United Kingdom
